# Nanoconfinement-Induced Structures in Chiral Liquid Crystals

**DOI:** 10.3390/ijms140917584

**Published:** 2013-08-28

**Authors:** Michael Melle, Madlona Theile, Carol K. Hall, Martin Schoen

**Affiliations:** 1 Stranski-Laboratorium für Physikalische und Theoretische Chemie, Technische Universität Berlin, Straße des 17. Juni 135, Berlin 10623, Germany; E-Mails: madlona.theile@campus.tu-berlin.de (M.T.); martin.schoen@tu-berlin.de (M.S.); 2 Department of Chemical and Biomolecular Engineering, North Carolina State University, 911 Partners Way, Raleigh, NC 27695, USA; E-Mail: hall@ncsu.edu (C.K.H.)

**Keywords:** liquid crystal, chirality, cholesteric and blue phases, confinement, Monte Carlo simulation

## Abstract

We employ Monte Carlo simulations in a specialized isothermal-isobaric and in the grand canonical ensemble to study structure formation in chiral liquid crystals as a function of molecular chirality. Our model potential consists of a simple Lennard-Jones potential, where the attractive contribution has been modified to represent the orientation dependence of the interaction between a pair of chiral liquid-crystal molecules. The liquid crystal is confined between a pair of planar and atomically smooth substrates onto which molecules are anchored in a hybrid fashion. Hybrid anchoring allows for the formation of helical structures in the direction perpendicular to the substrate plane without exposing the helix to spurious strains. At low chirality, we observe a cholesteric phase, which is transformed into a blue phase at higher chirality. More specifically, by studying the unit cell and the spatial arrangement of disclination lines, this blue phase can be established as blue phase II. If the distance between the confining substrates and molecular chirality are chosen properly, we see a third structure, which may be thought of as a hybrid, exhibiting mixed features of a cholesteric and a blue phase.

## 1. Introduction

Chirality is a symmetry property that is omnipresent, not only in many fields of science [1], but also in everyday life. A simple and intuitive example of a chiral object that everybody is familiar with is the human hand. Here, chirality gives rise to the fact that it is impossible to put a right-handed glove over the left hand and *vice versa*. The first more general definition of the term “chirality” was given by Lord Kelvin [2] who stated: “I call any geometrical figure or group of points, *chiral*, and say that it has chirality if its image in a plane mirror, ideally realized, cannot be brought to coincide with itself.”

There is a vast number of examples from all scientific disciplines that comport with Lord Kelvin’s definition of chirality. To name just a few, in mathematics, the well-known Möbius strip (see, for example, Figure 5.6 in [3]) is an example of a two-dimensional chiral structure, because no combined rotation and reflection operations exist that would transform one conformation of the Möbius strip into its mirror image. In physics, one encounters chirality in the propagation of circularly polarized light, where the temporal evolution and direction of the electric-field vector propagates along a helix in space. In chemistry, many molecules exhibit chirality. Here, pairs of molecules with chemically identical composition, but molecular structure of different handedness (*i.e*., enantiomers), often have quite disparate physico-chemical properties, such as the direction into which they rotate polarized light. However, regardless of the specific example and the scientific discipline from which it was taken, chirality, in general, can be traced back to the lack of roto-inversion symmetry of the specific phenomenon or object under consideration. In other words, for a molecule to be chiral, there must not exist any plane that divides the molecule into a pair of mirror images.

A particularly interesting class of materials often exhibiting chirality are liquid crystals. Here, it is the combined effect of molecular chirality and the formation of orientationally ordered, but positionally disordered, nematic or smectic mesophases, which gives rise to new structures, such as cholesteric or blue phases, to which the present article is devoted. Both cholesteric and blue phases are characterized by molecular helices in one or more spatial directions. The pitch length characterizing these helical structures ranges typically from a few hundred nanometers to a few microns in length, such that chiral liquid crystals exhibit interesting optical properties over a wide range of wavelengths from the ultraviolet all the way to the visible part of the electromagnetic spectrum [4,5]. Because of the structural complexity of chiral liquid crystals [6], it is not surprising that these materials have already received quite a bit of attention, both from the experimental and from the theoretical point of view for quite some time [7].

In particular, blue phases exhibit specular reflections of visible light that can be controlled by external fields, such that these phases may be thought of as tunable photonic materials [8] with a wide range of applications. However, their thermal stability is quite limited (usually restricted to a very narrow temperature range of only a few K [5]), such that for applications of practical value, the broadening of the regime of thermal stability of blue phases is an indispensable prerequisite [9]. Factors that enhance the thermal stability of blue phases have been investigated by Zheng *et al.* [10], He *et al.* [11] and Yoshizawa *et al.* [12].

In the context of novel nanomaterials, the fabrication of three-dimensional nanostructures has been achieved recently by polymer templating blue phase I [13]. Hydrogen-bonded liquid crystals act as an optical shutter if exposed to DC voltage and may, thus, be used to modulate light effectively [14,15]. In nanostructured chiral liquid crystals, the Kerr effect (*i.e*., the variation of optical properties with an applied external electric field) may be employed to fabricate materials with interesting electro-optical properties [16]. The optical properties of cholesteric liquid crystals makes them attractive candidates for optical switches. For instance, at zero field, two stable states exist, which are referred to as “planar texture” and “focal conic structure” [17]. In the former state, these materials reflect circularly polarized light, whereas in the latter state, they scatter light in the forward direction. This makes bi-stable cholesteric phases also very interesting in display technology, because of their low power consumption and low cost of operation [18].

On the theory side, quite a bit of work has been invested to understand defects in liquid crystals and their topology [3]. The interest in defects is currently stimulated by the fact that they give rise to long-range elastic forces in colloidal particle systems imposed by liquid-crystal phases [19]. These forces may then be used to self-assemble colloids to form much larger supramolecular and highly ordered structures [20]. In particular, in chiral liquid crystal phases, defects give rise to disclination lines of quite a complex geometry [20].

The interaction potential of a nanocolloid with a $-\frac{1}{2}$ disclination line has been shown to provide a generic trapping mechanism for particles immersed in a blue phase [21]. Here, the calculation of disclination lines is based upon a minimization of a properly formulated variant of Landau-de Gennes’ theory. A very useful article reviewing the rich structural features of chiral liquid-crystalline phases was published a while ago by Kamien and Selinger [22]. Orientational frustration and the presence of disclination lines in chiral liquid crystals are intimately intertwined, as was noted quite some time ago by Sethna [23].

Structural richness in ordered chiral liquid-crystalline phases is also highlighted by Fukuda and Žumer, who observe that highly chiral nematic liquid crystal phases are capable of forming two-dimensional, so-called Skyrmion lattices, as a thermodynamically stable morphology [24]. Here, particle-like topological entities (*i.e*., Skyrmions) are forming in a continuous field that are also important in understanding the quantum Hall effect in two-dimensional electron gases, chiral ferromagnets or Bose-Einstein condensates. In an earlier study, the latter authors also observed novel disclination-line topologies in blue phase I under severe confinement conditions [25].

Almost all these previous theoretical studies have been based upon mesoscopic approaches, such as the Frank-Oseen elastic equations [26], whereas only a few studies to date employ molecular simulations, such as molecular dynamics or Monte Carlo (MC). This is because the helical structures forming in prototypical chiral liquid crystals are large on a molecular scale, meaning that very large systems have to be used in these simulations. This was out of reach, until fairly recently, because of a lack of sufficient computational power.

Quite a bit of simulation work had been carried out in the 1990s by Memmer and coworkers. They studied the temperature dependence of the pitch that characterizes cholesteric phases [27,28] based upon a Gay-Berne-type model fluid that they properly modified to account for the pairwise additive interactions between chiral molecules [27]. However, because of the much reduced power of computers at the time of writing those earlier papers, only very small systems were investigated, containing as few as 256 molecules. Because of this smallness only very limited conclusions about the molecular nature of helical structures could be drawn. In fact, Memmer reports a substantial system-size dependence in a later study [29]. As we will demonstrate below, much larger systems containing at least some 10,000 particles are required if one wishes to gain deeper insight into the rich structural features of chiral liquid crystals from a molecular perspective.

The remainder of our paper is organized as follows. In Section 2, we introduce our model system. Section 3 is given as a summary of some key theoretical concepts. In Section 4, we present our results which we summarize in concluding Section 5.

## 2. The Model

Our model consists of *N* liquid-crystal molecules (*i.e*., mesogens) under nanoconfinement conditions, such that we may decompose the total configurational energy into a fluid-fluid (ff) and into a fluid-substrate (fs) contribution, according to:

(1) $$\Phi (R,\hat{U})=\Phi_{\text{ff}}(R,\hat{U})+\Phi_{\text{fs}}(Z,\hat{U})$$

We introduce shorthand notation, *Z* ≡ {*z*_1_*, z*_2_*, . . . , z_N_*}, for the set of *z*-coordinates of the center-of-mass positions, ***R*** ≡ {***r***_1_*,****r***_2_*, . . . ,****r****_N_*}, of the *N* mesogens and for their orientations represented by the set of unit vectors, ***Û*** ≡ {***û***_1_*,****û***_2_*, . . . ,****û****_N_*}, where the caret is used throughout this paper to indicate a unit vector. More specifically:

(2) $$\Phi_{\text{ff}}(R,\hat{U})=\frac{1}{2}\sum_{i=1}^{N}\sum_{j\neq i}^{N}\phi_{\text{ff}}\;(r_{ij},{\hat{u}}_{i},{\hat{u}}_{j})$$

where ***r****_ij_* ≡ ***r****_i_*−***r****_j_* is the distance vector between the centers of particles *i* and *j*, assuming the pairwise additivity of their interactions. In Equation (2):

(3) $$\phi_{\text{ff}}(r_{ij},{\hat{u}}_{i},{\hat{u}}_{j})=4{ɛ}_{\text{ff}}\;\left[{\left(\frac{\sigma }{r_{ij}}\right)}^{12}-{\left(\frac{\sigma }{r_{ij}}\right)}^{6}\;\{1+\Psi \;({\hat{r}}_{ij},{\hat{u}}_{i},{\hat{u}}_{j})\}\right]$$

where *σ* denotes the “diameter” of a spherical (Lennard-Jones) reference particle, *ɛ*_ff_ is the potential well depth, *r_ij_* = *|****r****_ij_|* and ***û****_i_* and ***û****_j_* are the orientations of the interacting molecules, *i* and *j*. The orientation dependence of the intermolecular interactions is accounted for by the function:

(4) $$\Psi =5{ɛ}_{1}P_{2}({\hat{u}}_{i}\cdot {\hat{u}}_{j})+5{ɛ}_{2}[P_{2}({\hat{r}}_{ij}\cdot {\hat{u}}_{i})+P_{2}({\hat{r}}_{ij}\cdot {\hat{u}}_{j})]+{ɛ}_{3}[({\hat{u}}_{i}\times {\hat{u}}_{j})\cdot {\hat{r}}_{ij}]\;({\hat{u}}_{i}\cdot {\hat{u}}_{j})$$

where $P_{2}(x)=\frac{1}{2}(3x^{2}-1)=P_{2}(-x)$ is the second Legendre polynomial and *ɛ*_1_ = 0.04 = −*ɛ*_2_*/*2 are anisotropy parameters, which we fix to these values throughout this work. The pseudoscalar on the far right side of Equation (4) introduces the chirality of the intermolecular interactions, where the absolute value of the chirality parameter, *ɛ*_3_, determines the amount of chirality, while its sign defines the handedness of the chiral molecules. The most preferred configuration for two achiral molecules (*i.e*., *ɛ*_3_ = 0) is a side-side configuration (***û****_i_* · ***r̂****_ij_* = ***û****_j_* · ***r̂****_ij_* = 0) where the orientations are parallel or antiparallel to each other ( $|{\hat{u}}_{i}\cdot \hat{u_{j}}|=1$). For nonvanishing values of *ɛ*_3_, the preferred conformation of a pair of mesogens is still the side-by-side one, but now, with slightly tilted orientations, where the preferred tilt angle depends on the magnitude of *ɛ*_3_. Clearly, Ψ remains unaltered if one changes the sign of ***û****_i_* and/or ***û****_j_*, thus resembling the head-tail symmetry characteristic of many mesogens [30].

The achiral version of the interaction potential was suggested originally by Hess and Su some time ago [31]. The model has been demonstrated subsequently [19,32–38] to be capable of reproducing properties of liquid crystals in a variety of contexts, ranging from the formation of nematic phases [32–35] to the development of defect topologies arising near the surface of spherical colloidal particles immersed in a nematic liquid-crystal host phase [19]. The introduction of chirality through the pseudoscalar on the far right side of Equation (4) follows in spirit a suggestion by Memmer *et al.* [27] for the classical Gay-Berne model [39].

Chiral, as well as achiral liquid crystals are experimentally usually investigated under confinement, often between smooth parallel walls that may prefer a specific orientational alignment of the mesogens with respect to the surface plane. They can serve to manipulate the preferred global orientation, due to specific anchoring at the solid surfaces, which then allows one to fix the orientation of the global director in the nematic phase. “Anchoring” refers to an energetic discrimination of preferred (or undesired) molecular orientations, as we will explain in some more detail shortly. In the present study, we confine the chiral liquid crystal to a nanoscopic slit-pore with atomically smooth substrate surfaces to make contact with the geometry of a typical experimental setup. The fluid-substrate contribution to the total configurational potential energy in Equation (1) may then be expressed as:

(5) $$\Phi_{\text{fs}}(Z,\hat{U})=\sum_{k=1}^{2}\sum_{i=1}^{N}\phi_{\text{fs}}(z_{i},{\hat{u}}_{i})$$

where the fluid-substrate interaction potential is given by the Yukawa-like expression:

(6) $$\phi_{\text{fs}}^{\left(k\right)}={ɛ}_{\text{fs}}\;\left[a_{1}\;{\left(\frac{\sigma }{\Delta z_{i}}\right)}^{10}-a_{2}\frac{\text{exp }(-\eta |\Delta z_{i}|)}{|\Delta z_{i}|}g^{\left(k\right)}({\hat{u}}_{i})\right]$$

Here, *ɛ*_fs_ determines the depth of the attractive well and Δ*z_i_* = *z_i_± s*_z_*/*2, where the sign is chosen depending on whether the mesogen interacts with the lower (*k* = 1) or upper (*k* = 2) substrate located at −*s*_z_*/*2 and *s*_z_*/*2, respectively. The dimensionless parameters:

(7a) $$a_{1}=\frac{1+\eta \sigma }{9-\eta \sigma }$$

(7b) $$a_{2}=\frac{10\;\text{exp }(\eta \sigma )}{9-\eta \sigma }$$

are unique functions of the screening length, *η*^−1^. They are introduced to guarantee that the location of the minimum of the fluid-substrate potential, *z*_min_, defined by:

(8) $${\frac{\text{d}\phi_{\text{fs}}}{\text{d}z_{i}}|}_{z_{\text{min}}}=0$$

remains fixed and that the depth of the attractive well:

(9) $$\phi_{\text{fs}}\;(z_{\text{min}})=-{ɛ}_{\text{fs}}$$

is preserved as one varies the range of fluid-substrate attraction. However, in this work, we employ a fixed short-range, but sufficiently strong fluid-substrate attraction characterized by *ησ* = 1.0 and *ɛ*_fs_*/ɛ*_ff_ = 3.0.

Fluid-substrate attractions are “switched on/off” by the anchoring functions, 0 ≤ *g*^(^*^k^*^)^( ***û****_i_*) ≤ 1, depending on the orientation of a mesogen with respect to the substrate plane. Hence, the anchoring functions are introduced to discriminate energetically between undesirable and desirable orientations. In the present study, we employ:

(10a) $$g^{\left(1\right)}={\left({\hat{u}}_{i}\cdot {\hat{e}}_{\text{x}}\right)}^{2}$$

(10b) $$g^{\left(2\right)}={\left({\hat{u}}_{i}\cdot {\hat{e}}_{\text{x}}\right)}^{2}+{\left({\hat{u}}_{i}\cdot {\hat{e}}_{\text{y}}\right)}^{2}$$

where the unit vector, ***ê****_α_*, is pointing along the *x*- or *y*-axis of the Cartesian coordinate system. Hence, mesogens located in the immediate vicinity of the lower substrate will align preferentially with the *x*-axis, whereas mesogens near the upper substrate will align their longer axes with the *x*–*y* plane without preference for any orientation on the unit circle. We termed the former anchoring scenario “directional” and refer to the latter as “planar” [35]. According to Jerôme, directional anchoring is referred to as “monostable”, whereas planar anchoring constitutes a “degenerate” anchoring scenario, because an infinite number of orientations exist on the unit circle that are energetically favorable and equivalent [40]. Employing hybrid [*g*^(1)^≠ *g*^(2)^, see Equation (10)] rather than homogeneous anchoring (*i.e*., *g*^(1)^ = *g*^(2)^) is advantageous for reasons to be explained in Section 4.1.

## 3. Theoretical Background

In this work, we consider a liquid crystal composed of *N* mesogens confined to a nanoscopic slit-pore of volume *V* = *As_z_*, where *s_z_* is the distance between the pore walls located along the *z*-axis of a space-fixed Cartesian coordinate system, *A* = *s_x_s_y_* is the wall area and *s_α_* (*α* = *x, y*) is the linear extent of the pore wall in the *α*-direction. The liquid crystal is maintained at constant *s_z_*, temperature, *T*, and fixed transverse pressure, $P_{\|}\equiv \frac{1}{2}(P_{xx}+P_{yy})$, where *P_αα_* (*α* = *x, y*) is a diagonal component of the pressure tensor, **P**. Under these conditions, thermodynamic equilibrium states correspond to minima of a generalized Gibbs potential, 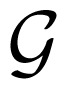, whose exact differential is given by:

(11) $$\text{d}G=-S\text{d}T+\mu \text{d}N+As_{z}\text{d}P_{\|}-AP_{zz}\text{d}s_{z}$$

where 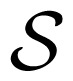 denotes entropy and *μ* chemical potential, such that {*N, T, P_||_, s_z_*}is the set of natural variables of 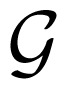.

At the molecular level, 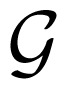 may be cast as [41]:

(12) $$G=-k_{\text{B}}T\;\text{ln }\chi$$

where *χ* = *χ* (*N, T, P_||_, s_z_* ) is the partition function and *k*_B_ is Boltzmann’s constant. As discussed elsewhere in detail (see pp. 33–70 in [42]):

(13) $$\chi =\sum_{s_{\text{x}},s_{\text{y}}}\text{exp }(-\beta P_{\|}As_{z})\;Q$$

where *β* ≡ 1*/k*_B_*T*, and 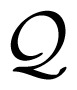 is the canonical-ensemble partition function in the classical limit. As discussed in detail by Gruhn and Schoen [43] (see also [44]):

(14) $$Q={\left(\frac{I}{m\Lambda^{5}}\right)}^{N}Z$$

where ℐ is the moment of inertia, *m* is the mass of a mesogen, Λ = (*h*^2^*/*2*πmk*_B_*T*)^1/2^ is the thermal de Broglie wavelength, *h* is Planck’s constant and the exponent, five, in the denominator of Equation (13) reflects the fact that we consider slightly elongated particles with three translational and two rotational degrees of freedom. In Equation (14):

(15) $$Z=\frac{1}{2^{N}N!}\int \int \text{d}R\text{d}\hat{U}\;\text{exp}[-\beta U(R,\hat{U})]$$

is the configuration integral and *U*(***R****,****Û***) is the total configurational potential energy. The factor, 1*/*2*^N^*, in front of the integral on the right side takes notice of the head-tail symmetry of the mesogens, that is, it corrects for double counting equivalent configurations characterized by ***û****_i_* and −***û****_i_*. The prefactor, 1*/N*!, arises on account of the indistinguishability of the mesogens in fluid phases. Combining Equations (13) and (15), we finally obtain:

(16) $$\chi =\sum_{s_{\text{x}},s_{\text{y}}}\text{exp }(-\beta P_{\|}As_{z})\;Q$$

Thus, from Equations (11), (12) and (16), properties, such as *μ* or *P_zz_*, can, in principle, be computed as ensemble averages in the generalized isothermal-isobaric ensemble defined through these three equations.

In some cases, the simulations have not been carried out in the specialized isothermal-isobaric ensemble introduced above, but in the grand canonical ensemble instead. In this case, the relevant thermodynamic potential is the grand potential:

(17) $$\text{d}\Omega =-S\text{d}T-N\text{d}\mu +P_{\|}s_{z}\text{d}A-AP_{zz}\text{d}s_{z}$$

and Equation (12) is replaced by:

(18) $$\Omega =-k_{\text{B}}T\;\text{ln }\Xi$$

where the grand canonical partition function is given by:

(19) $$\Xi =\sum_{N}\text{exp }(N\beta \mu )\;Q$$

Hence, in the grand canonical ensemble, a thermodynamic equilibrium state is characterized by the set, {*T, μ, A, s_z_* }, of natural variables of Ω.

In this work, we wish to analyze the local orientational order forming in a confined chiral liquid crystal for which we introduced the intermolecular interaction potentials already in Section 2. A suitable quantitative measure of local orientational order is provided through the local alignment tensor, which we define as:

(20) $$Q\;(r)\equiv \frac{1}{2\rho \;(r)}\sum_{i=1}^{N}\langle [3{\hat{u}}_{i}\;(r_{i})⊗{\hat{u}}_{i}\;(r_{i})-1]\;\delta \;(r-r_{i})\rangle$$

where *ρ* (***r***) is the local density, **1** is the unit tensor, *δ* (***r*** − ***r****_i_*) is the Dirac *δ*-function and the operator, “⊗”, denotes the tensor product. Angular brackets in Equation (20) indicate an ensemble average taken either in the specialized isothermal-isobaric or in the grand canonical ensemble. The local alignment tensor can be represented by a real, symmetric, traceless, second-rank 3 *×* 3 matrix and satisfies the (local) eigenvalue equation:

(21) $$Q\;(r)\;\hat{n}\;(r)=\lambda \;(r)\;\hat{n}\;(r)$$

Because **Q**(***r***) is a second-rank tensor, Equation (21) has as a solution three (local) eigenvalues, *λ*_−_ (***r***) *< λ*_0_ (***r***) *< λ*_+_ (***r***), and associated eigenvectors, ***n̂***_−_ (***r***), ***n̂***_0_ (***r***) and ***n̂***_+_ (***r***). We follow standard practice for the non-local analog of **Q**(***r***), *define*, as a local nematic order parameter, the largest eigenvalue and take the associated eigenvector as the local nematic director [45]. To ease the notational burden, we shall henceforth drop the subscript on both *λ*_+_ (***r***) and ***n̂***_+_ (***r***). To determine *λ* (***r***) and ***n̂*** (***r***) numerically, we employ the Jacobi transformation technique described in detail in the book by Press *et al.* [46].

Another quantity of interest is the pressure tensor, **P**. As pointed out in the Appendix of [34], one may employ the hypervirial theorem (see Appendix E.2 of [44]) and write:

(22) $$P=\rho k_{\text{B}}T1+\frac{1}{s_{z}}\langle \frac{1}{A}\sum_{i=1}^{N}r_{i}⊗F_{i}\rangle$$

where *ρ* is the number density and ***F****_i_* is the total force exerted on mesogen *i*. Focusing only on the diagonal components, *P_xx_* and *P_yy_*, one finds, after some straightforward algebra [34]:

(23) $$\begin{array}{l} P_{\alpha \alpha }=\rho k_{B}T-\frac{24ɛ}{s_{z}}\langle \frac{1}{A}\sum_{i=1}^{N}\sum_{j\neq i}^{N}\left[{\left(\frac{\sigma }{r_{ij}}\right)}^{12}-\frac{1}{2}{\left(\frac{\sigma }{r_{ij}}\right)}^{6}\;[1+\Psi \;({\hat{r}}_{ij},{\hat{u}}_{i},{\hat{u}}_{j})]\right]\;{\hat{r}}_{ij}^{\left(\alpha \right)}{\hat{r}}_{ij}^{\left(\alpha \right)}\rangle \\ -\frac{30ɛ{ɛ}_{2}}{s_{z}}\langle \frac{1}{A}\sum_{i=1}^{N}\sum_{j\neq i}^{N}{\left(\frac{\sigma }{r_{ij}}\right)}^{6}\{({\hat{u}}_{i}\cdot {\hat{r}}_{ij})\;{\hat{u}}_{i}^{\left(\alpha \right)}{\hat{r}}_{ij}^{\left(\alpha \right)}+({\hat{u}}_{j}\cdot {\hat{r}}_{ij}){\hat{u}}_{j}^{\left(\alpha \right)}{\hat{r}}_{ij}^{\left(\alpha \right)}-[{\left({\hat{u}}_{i}\cdot {\hat{r}}_{ij}\right)}^{2}-{\left({\hat{u}}_{j}\cdot {\hat{r}}_{ij}\right)}^{2}]{\hat{r}}_{ij}^{\left(\alpha \right)}{\hat{r}}_{ij}^{\left(\alpha \right)}\}\rangle \\ -\frac{4ɛ{ɛ}_{3}}{s_{z}}\langle \frac{1}{A}\sum_{i=1}^{N}\sum_{j\neq i}^{N}{\left(\frac{\sigma }{r_{ij}}\right)}^{6}({\hat{u}}_{i}\cdot {\hat{u}}_{j})\;\left[{\left({\hat{u}}_{i}\times {\hat{u}}_{j}\right)}^{\left(\alpha \right)}{\hat{r}}_{ij}^{\left(\alpha \right)}-({\hat{u}}_{i}\times {\hat{u}}_{j})\cdot {\hat{r}}_{ij}{\hat{r}}_{ij}^{\left(\alpha \right)}{\hat{r}}_{ij}^{\left(\alpha \right)}\right]\rangle \end{array}$$

where superscript ^(^*^α^*^)^ (*α* = *x, y*) refers to the *α*-component of the corresponding vector. Chirality enters the expression through the function, Ψ, on the first line of Equation (23) and through the term proportional to the chirality coupling constant, *ɛ*_3_, on the last line of that equation. Monitoring *P_xx_* and *P_yy_* independently is useful to validate the simulations to be discussed below, because their values should agree with the input value, *P_||_*, in the isothermal-isobaric ensemble. However, in case the side lengths, *s_x_* and *s_y_*, of the simulation box are coupled (*i.e*., within one MC cycle, there is an attempt to change the area, *A* = *s_x_s_y_*, rather than *s_x_* and *s_y_* individually) their actual values may differ. The origin of this difference is anisotropy induced by the directional anchoring at the walls. However, as discussed elsewhere [19], it is sufficient if the arithmetic mean, *P_||_*, coincides with the input value, a condition satisfied in this work at all times. Moreover, the deviation between either *P_xx_* or *P_yy_* and *P_||_* rarely exceeds 0.01 in all cases. Therefore, the chosen value of *P_||_* = 1.80 *σ*^3^*/ɛ*_ff_ exceeds this deviation by more than two orders of magnitude.

## 4. Results and Discussion

### 4.1. Numerical Details

We employ Monte Carlo (MC) simulations in the specialized isothermal-isobaric and in the grand canonical ensembles introduced briefly in Section 3. Because our intermolecular potentials are short range [see Equations (2) and (6)] we employ periodic boundary conditions at the planes located at *x* = *±s_x_/*2 and *y* = *±s_y_/*2 (assuming the origin of the Cartesian coordinate system to be located at the center of the simulation box). In the *z*-direction, where the solid substrates are separated by a fixed distance, *s*_z_, the implementation of hybrid anchoring [see Equation (10)] is advantageous in avoiding spurious stresses if the ratio of 2*s_z_/p* is non-integer, where *p* denotes the pitch length characterizing chiral structures forming in that direction. This is because the anchoring function in Equation (10a) serves to align mesogens with the *x*-axis in the vicinity of the upper substrate. Because of the degenerate nature of the anchoring scenario at the lower substrate, the planar alignment of the mesogens allows a helix forming in the *z*-direction to relax, even if *p/*2*s_z_* assumes non-integer values. Moreover, we create a static situation, where the helix’s orientation is fixed at one point along the *z*-axis, due to directional alignment at one wall. In other words, the helix stays put along the *z*-axis, which allows us to interchange summation and averaging in Equation (20) and, therefore, facilitates the analysis of our system. In addition, the substrate causes the *z*-direction to be distinct from the other two and induces a helical structure in that direction in all our simulations.

Because we are not interested in investigating any specific chiral liquid crystal, we express our quantities of interest in terms of “reduced” (*i.e*., dimensionless) units. For example, length is given in units of *σ*, energy in units of *ɛ*_ff_, temperature in units of *ɛ*_ff_*/k*_B_ and pressure in units of *ɛ*_ff_*/σ*^3^.

For the anisotropy parameters chosen throughout this work [see Section 2, Equation (4)] the aspect ratio of the mesogens was determined to be 1.26 [34], taking the ratios of zeros of *ϕ*_ff_ (***r****_ij_,****û****_i_,****û****_j_*) for the side-side (***û****_i_* · ***r̂****_ij_* = 0) and end-end configurations (*|****û****_i_* · ***r̂****_ij_|* = 1) of a pair of parallel aligned mesogens (*|****û****_i_* · ***û****_j_|* = 1) as a definition of their aspect ratio. This rather small value turns out to be advantageous, as it supports fast equilibration of the simulation sample. In combination with the simplicity of the model, it allows us to study fairly large systems containing several tens of thousands of molecules.

In the isothermal-isobaric ensemble MC simulations, we take *P_||_* and *T* to be constant at values of 1.80 and 0.95, respectively. Under these conditions, the achiral bulk version of our model liquid crystal (*ɛ*_3_ = 0) is sufficiently deep in the nematic phase, as reflected by the relatively high value of the global nematic order parameter, *λ* ≈ 0.7. In the grand canonical MC simulations, we chose *μ* = −9.588, which corresponds to *P_||_* ≃ 1.77 [computed via Equation (23)], so that the thermodynamic equilibrium states considered in both ensembles are comparable.

Our results are based upon systems comprising between 5, 000 and 40, 000 mesogens and runs consisting of about 5 *×* 10^4^ MC cycles that have been carefully equilibrated using a similar number of cycles prior to sampling any data. In the specialized isothermal-isobaric ensemble, a MC cycle consists of *N* random displacements or rotations followed by one attempt to change the size of the computational cell in the *x*- and *y*-directions. Displacement or rotation of a mesogen are both attempted with equal probability, where the size of the displacement cube centered on a mesogen’s center-of-mass position and the angle increment of rotation are adjusted during the simulation to guarantee an overall acceptance of 40%–70% of both attempted moves. In the grand canonical simulations, an MC cycle consists of *N*′ random displacements or rotations, followed by *N*′ attempts to either create a new mesogen at a randomly chosen position in the system and with a randomly chosen orientation or to destroy one of the already existing ones. Both creation and destruction are also attempted with equal probability. Because the number of mesogens in the system will generally vary as a result of creation and destruction attempts, *N*′ is the number of mesogens present in the simulation cell at the beginning of a new MC cycle. In our simulations, creation and destruction attempts are accepted, with a ratio of 2.2 *×* 10^−4^. In both ensembles, we employ the standard generalized Metropolis algorithms described in Chapter 5 of [42]. These algorithms allow one to realize numerically Markov processes that generate distributions in configuration space proportional to exp{−*β* [*U*(***R****,****Û***) + *P_||_A* − *Nβ*^−1^ ln*A*]} and exp{−*β* [*U*(***R****,****Û***) − *μN*] − ln*N*! − 5*N* ln(Λ*m/*ℐ)} in the specialized isothermal-isobaric and in the grand canonical ensemble, respectively.

To save computer time and because our fluid-fluid interaction potential [see Equation (2)] is short-range, we employ a potential cutoff of *r*_c_ = 3.0. No corrections are applied for neglected interactions beyond *r*_c_. Moreover, *u*_ff_ (*r*_c_) remains unshifted with respect to *u*_ff_ = 0. For the fluid-fluid interactions, we utilize a combination of a link-cell and a conventional Verlet neighbor list, as described in the book by Allen and Tildesley [47] to further speed up the simulations. This latter list includes as neighbors all mesogens whose centers-of-mass are separated by a distance, *r_n_* = 3.8, from that of a reference mesogen. By employing the two smooth walls, we make the *z*-direction distinct from the other two. In order to investigate the local nematic order and the local director parallel to the wall, we divide our system along the *z*-axis into 200 equally-sized slabs of volume *s_x_s_y_δz*, where *δz* is the thickness of each slab. For mesogens located in these slabs, Equations (20) and (21) are then solved as described above to obtain *λ* (***r***) and ***n̂*** (***r***). During our investigations of complex chiral phases, it turned out to be practical and suitable to apply the same procedure in the *x* and *y* direction.

### 4.2. Structure of Ordered Phases

**Figure 1:**
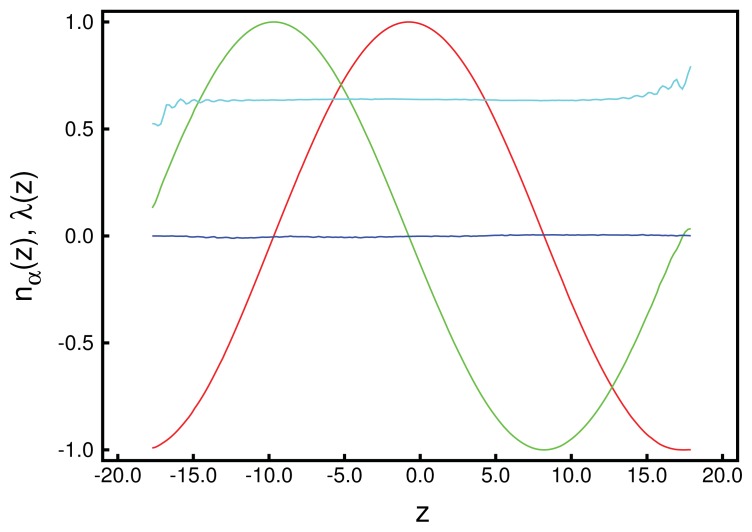
Plots of Cartesian components of the local director field, ***n̂*** (*z*), as a function of position along the *z*-axis perpendicular to the plane of the solid substrates separated by *s_z_* = 35; ( 
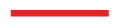
) *n_x_* (*z* ), ( 
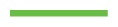
) *n_y_* (*z* ), ( 
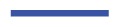
) *n_z_* (*z* ). Also shown is the local nematic order parameter, *λ* (*z* ) ( 
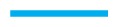
).

We begin the structural analysis with a discussion of the local director for a system at relatively low chirality, *ɛ*_3_ = 0.14. Unfortunately, in general, ***n̂*** (***r***) is a three-dimensional vector field depending on the three-dimensional vector, ***r***, and is, therefore, impossible to display in full. However, utilizing the fact that the *z*-direction is distinct in our system, we begin by analyzing ***n̂*** (*z*) in Figure 1. Data plotted in that figure illustrate the accuracy with which these curves can be obtained, provided *λ* (*z*) is sufficiently large. From the plot of the local order parameter, *λ* (*z*), it is evident that the entire confined liquid crystal exhibits a substantial nematic order in the present case. One also notices a slight asymmetry in that *λ* (*z*) is slightly lower at the lower substrate compared with its value at the upper substrate. This is because of the hybrid anchoring employed to generate these data [see Equations (10)]. The monostable directional anchoring along the *x*-axis causes a somewhat higher nematic order parameter compared with the degenerate planar one, because the latter is characterized by an infinite number of easy axes on the unit circle, whereas a mesogen not aligning with the *x*-axis receives an energy penalty. The order parameter close to the planar-anchoring wall is even lower than in the bulk-like portion of the confined liquid crystal centered on *z* = 0.

A mesogen located in that bulk-like region is surrounded by nearest neighbors in all three spatial directions. The orientation of these neighbors is tilted with respect to the reference mesogen, because at *ɛ*_3_ ≠ 0, this configuration is energetically favorable. If, on account of thermal fluctuations, the tilt angle deviates from its optimum value (at *T* = 0), the reference mesogen receives an energy penalty from *all* its neighbors. Mesogens located in the contact layer (*i.e*., the molecular layer in the immediate vicinity of a solid substrate) lack nearest neighbors in the direction towards the substrate. Thus, the number of nearest neighbors is reduced for these mesogens compared with those near the center of the simulation cell. As a result, the total energy penalty is smaller if the tilt angle between pairs of mesogens near the solid substrates deviates from the optimum value, which offers a possibility for somewhat larger orientational fluctuations.

**Figure 2:**
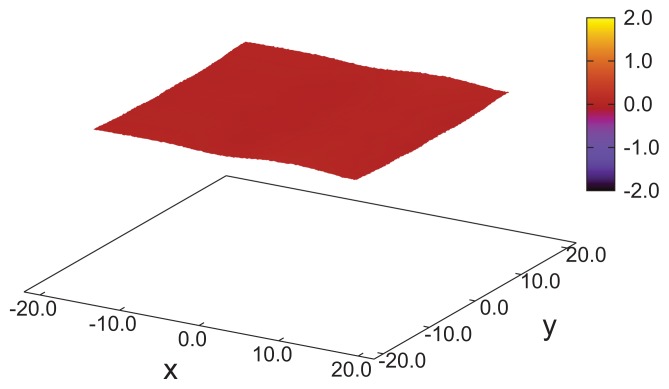
Plot of the sum, *n_z_* (*x*) + *n_z_* (*y*), indicated by the attached color bar, as a function of position in the *x*–*y* plane for a cholesteric phase forming at *ɛ*_3_ = 0.14 (see, also, Figure 1). Data plotted have been averaged over *z*.

Looking next at the associated local director, ***n̂*** (*z*), we realize that it varies periodically with *z*. Moreover, its spatial variation along the *z*-axis can be well described by the vector:

(24) $$\hat{n}\;(z)=\left(\begin{array}{c} \text{sin }(2\pi z/p)\\ \text{cos }(2\pi z/p)\\ 0 \end{array}\right)$$

where *p* is the pitch of the periodically varying structure. This also means that in accordance with our data in Figure 1, the director, ***n̂*** (*z*), rotates perpendicular to the *z*-axis (*i.e*., parallel to the walls), such that its *z*-component vanishes. By fitting Equation (24) to the MC data, we obtain a pitch length, *p* ≃ 36.7, which exceeds the substrate separation of *s_z_* = 35 only marginally. Hence, the periodic structure along the *z*-axis does not fit *s*_z_ perfectly. Therefore, the plots of *n_x_* (*z*) and *n_y_* (*z*) in Figure 1 are indicative of a typical cholesteric phase characterized by a single helix rotating around the *z*-axis. Another characteristic feature of the cholesteric phase is that components, such as *n_z_* (*x*) and *n_z_* (*y*), of ***n̂*** (***r***) vanish, that is, there is in-plane homogeneity of the director field along the *z*-axis. This in-plane homogeneity is nicely illustrated by the plot in Figure 2.

**Figure 3:**
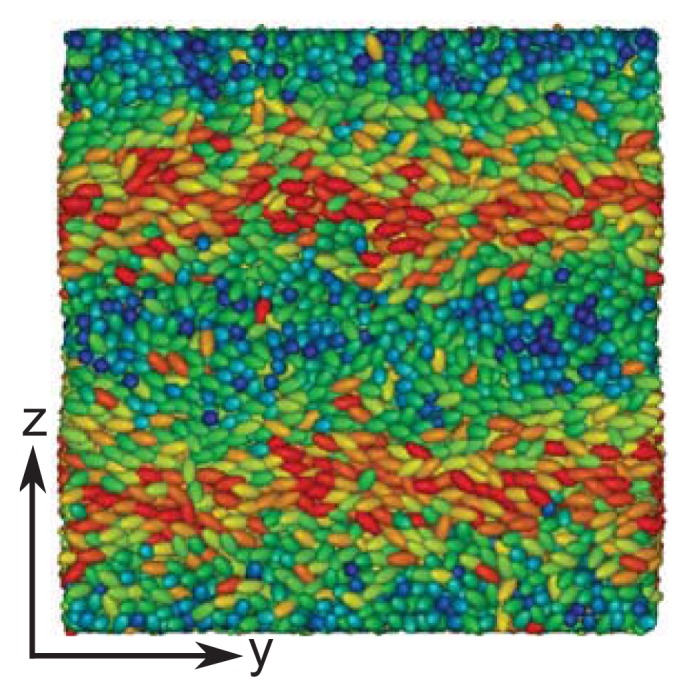
Side view of a “snapshot” of a typical configuration in the cholesteric phase, where the *z*-axis is vertical. The elongation of the mesogens is exaggerated to enhance the visibility of specific orientations. The color code is used to distinguish between molecules pointing in the *x*-direction (blue, out of the paper plane) and those whose orientation deviates maximally, *i.e*., by 90*°* (red). Other colors indicate that the particular mesogen is pointing in other directions, such that 0 *< |****û****_i_* · ***ê***_x_*| <* 1. The confining substrates at the top and bottom of the plots are not shown. The plot has been generated for *ɛ*_3_ = 0.14 with *N* = 40*,* 000 mesogens.

These structural features are further corroborated by the plot of a typical “snapshot” of a configuration in the cholesteric phase taken from our MC simulations (see Figure 3). As one can see, regions can be identified that are more or less homogeneously colored. The color of these regions varies only as one moves along the *z*-axis (that is, vertically), but remains nearly the same at fixed *z* in the *x*–*y* plane. The alternating blue and red colored regions indicate that along the *z*-axis, confinement by the solid substrates is such that nearly a full pitch of the helix is accommodated. That *s_z_* = 35 is slightly too small to accommodate a full pitch can be seen from the extent of the blue colored regions at the bottom and top of the plot, which is slightly thinner for the former compared with the latter. This visual observation is fully in line with the value of *p* ≃ 36.7 extracted by fitting Equation (24) to the curves shown in Figure 1.

**Figure 4:**
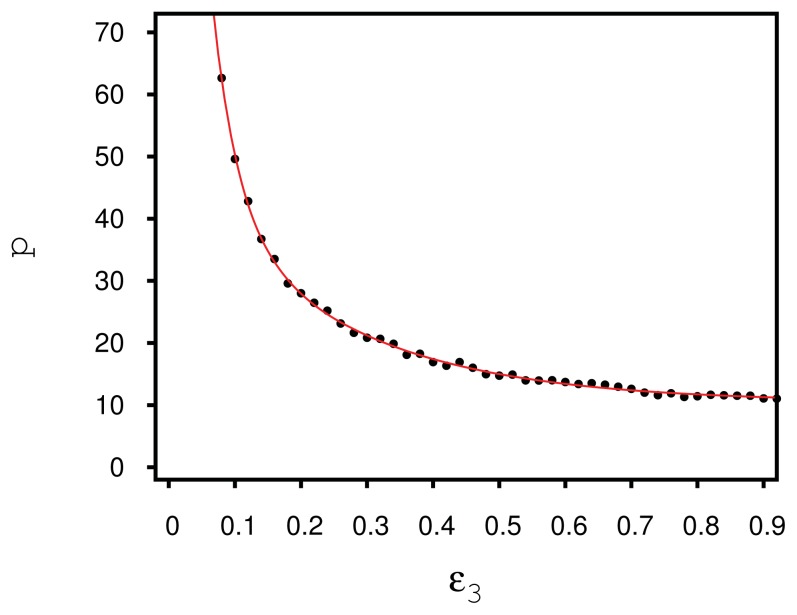
Pitch length as a function of the chiral coupling constant, *ɛ*_3_. The red line is intended to guide the eye. The transition from the cholesteric to the blue phase occurs at *ɛ*_3_ = 0.2.

In fact, closer scrutiny reveals that the helical structures formed by the liquid crystal depend on the chirality coupling parameter, *ɛ*_3_. Simulations in the specialized isothermal-isobaric ensemble were performed with 15,000 mesogens and a substrate distance of *s_z_* = 26 between the hybrid aligning substrates, so that the helix forming between them is not exposed to any spurious strain in the *z*-direction. The number of mesogens and the substrate distance are chosen, such that the simulation box is roughly cubic. Starting with a low value of *ɛ*_3_ = 0.08, several simulations are performed, where the chirality is slightly increased by Δ*ɛ*_3_ = 0.02 between subsequent simulations; in each of these simulations, the nematic director is computed as a function of *z*. Employing Equation (24), the pitch length can be determined over a wide chirality range, as demonstrated in Figure 4. The monotonic decay of *p* with increasing *ɛ*_3_ can be attributed to the stronger twist between neighboring mesogens. For very high chiralities, we observe the pitch length to reach a plateau. For increasingly larger values of *ɛ*_3_, highly twisted structures are energetically favored. However, the formation of these structures eventually causes even nearest neighbor molecules to deviate strongly from a side-side conformation favored energetically by the terms proportional to *ɛ*_1_ and *ɛ*_2_ in Equation (4). Thus, the energetic gain in forming tilted conformations of pairs of molecules is counterbalanced by the energy penalty, due to increasingly larger deviations from these side-side arrangements.

**Figure 5:**
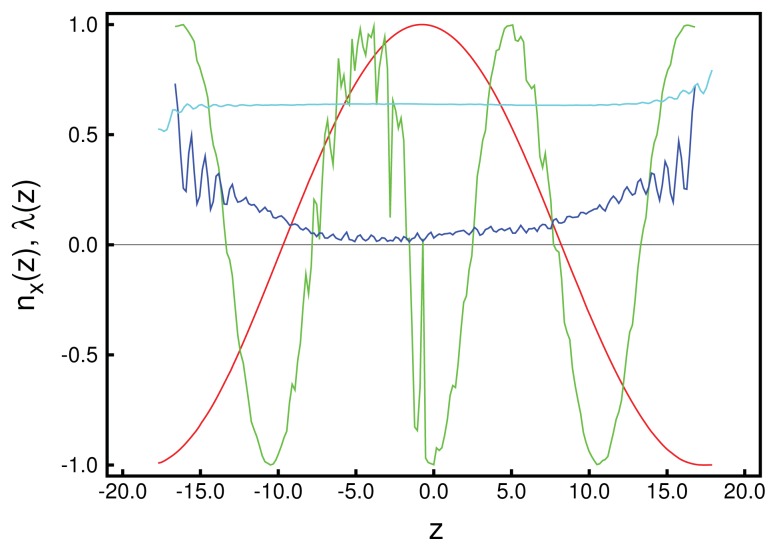
Components *n_x_* (*z*) as a function of position along the *z*-axis for ( 
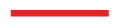
) *ɛ*_3_ = 0.14 (cf., Figure 3) and ( 
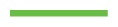
) *ɛ*_3_ = 0.90. In addition, the local nematic order parameter, *λ* (*z*), is shown for *ɛ*_3_ = 0.14 ( 
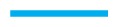
) and *ɛ*_3_ = 0.90 ( 
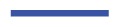
). For *ɛ*_3_ = 0.90, mesogens are directionally anchored at both substrate surfaces [see Equation (10a)], because the planar alignment leads to a decrease of the order, as explained in the text and, therefore, destabilizes the structure. Here, *s_z_* is chosen to coincide roughly with 3*p*. For *ɛ*_3_ = 0.14, hybrid anchoring is employed [see Equations (10)], as already explained.

Moreover, one notices that the local nematic order decreases with enhanced chirality. This can be seen from plots in Figure 5, where *λ* (*z*) around *z* = 0 and for *ɛ*_3_ = 0.90 decays to a value characteristic of an isotropic phase. However, the liquid crystal is not isotropic locally, but does indeed form a highly structured, morphologically distinct phase under these conditions, as we shall demonstrate shortly.

As we showed above in the cholesteric phase, mesogens are aligning with a local nematic director pointing in a direction somewhere in the *x*–*y* plane. The orientation of the director varies only as a function of *z*. This gives rise to the typical helical structure illustrated by the plots in Figures 1, 2 and 3. However, in each of the *x*–*y* planes along the *z*-axis, mesogens can preserve their parallel alignment, which is favored by the first two terms on the right side of Equation (4). In other words, provided *ɛ*_3_ is sufficiently small, nematic order can be preserved *locally*.

**Figure 6:**
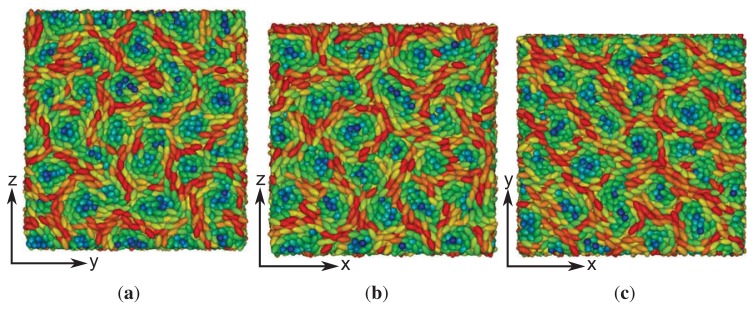
“Snapshot” of a typical configuration characteristic of a blue phase. Plots in (**a**)–(**c**) are side views, where the line of vision is along the *x*-, *y*- and *z*-axis, respectively, which are orthogonal to the paper plane in all three cases. Hence, mesogens colored in blue are aligned with the respective line of vision, whereas the orientation of mesogens colored in red is orthogonal to the line of vision. Plots have been generated for *ɛ*_3_ = 0.90.

Now, with increasing *ɛ*_3_, in-plane, twisted conformations of mesogens become energetically more favorable, which, in turn, destroys the local, in-plane nematic order of the cholesteric phase described before. In fact, under the present conditions, the liquid crystal exhibits the complex structure reminiscent of a blue phase. This can be seen from the snapshots presented in Figure 6, which illustrate the complex structures forming at sufficiently high values of the chirality coupling parameter, *ɛ*_3_.

In particular, one notices that in each of the three plots, regions of blue colored mesogens at the center exist. As one moves out of these regions from the center in any radial direction, the color of the mesogens changes from blue to green and, eventually, to red, as one reaches the circumference of a region in which the mesogens are aligned with the respective line of vision. This change in color reflects a change in orientation, where mesogens at the center of each region are aligned with the line of vision, whereas along the circumference, they are oriented in an orthogonal fashion with that respective line. Hence, this orientational change is characteristic of a double-twist alignment of the mesogens. Because the global topology of the three structures depicted in Figure 6 looks the same irrespective of the specific line of vision, we are dealing with a three-dimensional double-twist helical structure, which is characteristic of blue phases [48].

It seems somewhat surprising that the blue phase, which is isotropic *globally*, is still characterized by a small residual value of *λ* (*z*), as one can see from the plots in Figure 5. However, these small values of *λ* (*z*) should be perceived as a consequence of the finiteness of our system, an effect which is well understood and has been analyzed quantitatively for the achiral version of our model [37]. As before, in the cholesteric phase, *n_x_* (*z*) and *n_y_* (*z*) can be described by sine or cosine functions, indicating that there is a remaining director rotating in the *x*–*y* plane, but, now, with an almost vanishingly small order parameter, which also explains the relative noisiness of *n_x_* (*z*) in Figure 5. One can also see that as one approaches the walls of the slit-pore, the plot of *n_x_* (*z*) becomes much smoother. Nevertheless, the clearly visible periodicity of *n_x_* (*z*) allows us to determine the pitch length, *p*, reliably, even though the system at *ɛ*_3_ = 0.90 is optically isotropic in a global sense, as is expected for the blue phase [48]. Towards the substrates, nematic order increases, because of the directional anchoring that we apply at both substrate surfaces. Therefore, the present simulation of a blue phase was performed with directional anchoring at both walls, and the wall distance was estimated to give space for 3*p*.

The statistical accuracy of our data can be rationalized as follows. Taking ***û*** (***r***) as the local orientation of a single mesogen, we can express the local nematic order parameter alternatively as:

(25) $$\lambda (r)=\frac{1}{2}\int [3\;{\text{cos}}^{2}\;\vartheta \;(r)-1]\;P\;[\vartheta \;(r)]\;\text{d}\Omega$$

where cos *ϑ* (***r***) = ***û*** (***r***) · ***n̂*** (***r***) is the cosine of the angle between ***û*** (***r***) and the local nematic director, ***n̂*** (***r***), *P* [*ϑ* (***r***)] is the distribution of orientations of a mesogen with respect to ***n̂*** (***r***), because of the uniaxial symmetry of the mesogen, and dΩ is the solid angle. Clearly, *λ* (***r***) = 0 if *P* [*ϑ* (***r***)] is uniform. If, on the other hand, *P* [*ϑ* (***r***)] is strongly peaked, such that, for example, *P* [*ϑ* (***r***)] = *δ* [*ϑ* (***r***) − *π*], *λ* (***r***) = 1, where *δ* denotes the Dirac *δ*-function, in other words, if the variance of the distribution, *P* [*ϑ* (***r***)], about its mean is large, the associated local nematic order parameter, *λ* (***r***), will be small and *vice versa*. However, a large variance in *P* [*ϑ* (***r***)] implies a large variance of the local alignment tensor about its mean value and, therefore, a low statistical accuracy of ***n̂*** (***r***) obtained as a solution of Equations (20) and (21). Plots in Figure 5 support this line of argument.

**Figure 7:**
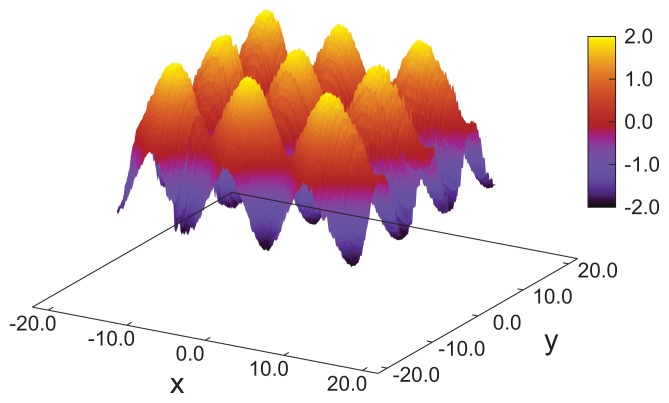
As Figure 2, but for *ɛ*_3_ = 0.90 in the blue phase.

As indicated by snapshots in Figure 6, blue phases show isotropic behavior in all three spatial directions. Based upon our results for the (components of) the director field in the *z*-direction, one anticipates rotating components of the director field in the *x*- and *y*-directions, as well. It is particularly noteworthy that in our simulations, the helical structures forming along all three directions (*x*, *y* and *z*) are always perpendicular to these axes. This is indeed a valid conclusion and can nicely be illustrated by plots of *n_z_* (*x*) + *n_z_* (*y*) in Figure 7. Consider that any arbitrary point on the given surface of the plot in that figure will follow sine curves as one moves along the *x*- *or y*-directions, reflecting the helical structure in those directions. Together with the plot of *n_x_* (*z*) in Figure 5, this indicates a regular double-twist helical structure in all three spatial dimensions characterized by a pitch of about ten molecular diameters. The regularity of the structure of the blue phase is consistent with the visual inspection of individual snapshots of configurations displayed in Figure 6. Nevertheless, the particle number needs to be chosen in such a way that an integer number of half-pitches can be formed, thereby avoiding the exposition of the entire helix to spurious strains otherwise caused by applying periodic boundary conditions at the ends of the simulation cell. The optimum system size has then been determined based upon the plot presented in Figure 4 and the ratio of 2*s_z_/p* that we decided to employ in the simulations.

We note in passing that observing the complex three-dimensional double-twist structure of a blue phase is not only a theoretical challenge, but also an experimental one. Indeed, the typical platelet structure of the blue phases seen experimentally is a result of a non-homogeneous crystallization [9], which is difficult to control.

**Figure 8:**
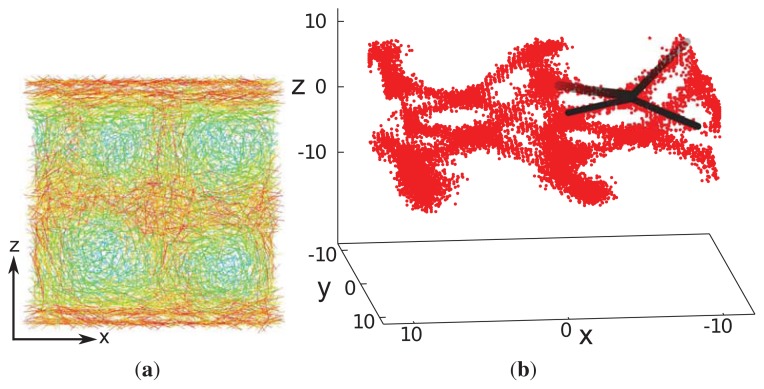
(**a**) “Snapshot” of blue phase II in which the double-twist helices (blue-green domains) exhibit a simple-cubic unit cell. To enhance the visibility, the widths of the mesogens have been arbitrarily reduced to zero in the plot. The line of vision is along the *y*-axis; (**b**) Disclination lines in three-dimensional space forming a network of tetrahedral geometry to be expected for blue phase II. Note that for an improved visualization of the complex geometrical arrangement of the disclination lines, the *z*-axis has been compressed relative to the *x*- and *y*-axes.

Nevertheless, we stress that the results obtained in this study clearly show that MC simulations of fairly large systems—if carried out with care—are capable of reproducing three-dimensional blue-phase structures comprising a number of pitches in each direction. However, up to this point, our results do not permit us to identify the specific blue phase (I, II or III) forming in our simulations. While blue phase I and II are characterized by a regular lattice of double-twist helices, blue phase III exhibits an amorphous structure. We, therefore, speculate that the presented structure might represent a blue phase III, which also is seen experimentally for systems with higher chirality [17]. However, the strong chirality and, therefore, the strong twist of the mesogens causes a more detailed analysis to be rather exhausting. To address this point, we amended our study by considering a system with smaller chirality, *ɛ*_3_ = 0.30, causing a larger pitch length and containing only *N* = 8*,* 550 molecules. This causes a larger pitch of *p* ≃ 21, such that the system can accommodate a single pitch in each spatial direction. The blue phase forming under these conditions is then identified through plots of ***n̂*** (*x* ), ***n̂*** (*y*) and ***n̂*** (*z* ). Accompanying snapshots of individual configurations exhibit double-twist helices forming in parallel with the *x*-, *y*- and *z*-axes indicating that the blue phase is not exposed to spurious strains (see Figure 8a). In each spatial direction, the double-twist helices are arranged corresponding to a simple-cubic unit cell, which is indicative of the structure anticipated for blue phase II.

This interpretation is further corroborated by an inspection of the associated disclination lines, which we define as regions in space with a nematic order parameter, *λ* (***r***) ≤ 0.30. Whereas this choice of *λ* (***r***) is admittedly somewhat arbitrary, it can be justified by the observation that *λ* ≈ 0.30 turns out to be the inflection point in plots of *λ versus* the thermodynamic field driving the isotropic-nematic phase transition in the achiral analogue of our model system [34]. As one can then see from Figure 8b, the disclination lines form a tetrahedral structure, as expected for blue phase II [20].

Up to this point we introduced the confining substrate merely as a technical means that allows us to determine the pitch length reliably and without interference from periodic boundary conditions. However, in parallel experiments, confining solid substrates are part of the standard setup. This prompted us to investigate the impact of the presence of these substrates on structural properties of the liquid crystal by varying *s_z_* in small steps of magnitude Δ*s_z_* = 0.1 between subsequent runs. In this part of our study, we employ a coupling parameter, *ɛ*_3_ = 0.24, which is slightly bigger than the one for which the system undergoes a transformation from a cholesteric to a blue phase, according to Figure 4. Whereas picking a certain chirality is obviously easy in our model system, one cannot easily do the same experimentally. The obvious reason is that in experimental systems, chirality is associated with a particular chemical structure and cannot be varied easily. However, the chirality of experimental systems can be altered by considering binary mixtures in which one component consists of chiral, the other one of achiral mesogens [10].

**Figure 9:**
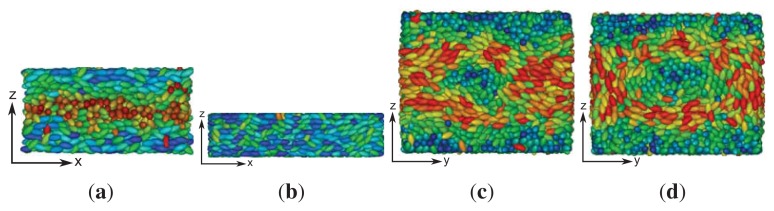
“Snapshots” of configurations of confined systems where both substrates cause directional alignment. The phase determining parameter is the substrate-substrate distance, which is 8.2 for the nematic phase in (**b**), 12.5 for the cholesteric phase in (**a**) and 21.3 for the confined phase in (**c**) and (**d**). (**c**) and (**d**) show the same system, but different cross sections, where the nematic director is pointing in the *y*- (part **c**) or *z*-direction (part **d**), respectively. All systems have been generated for *ɛ*_3_ = 0.24.

According to the plot in Figure 4, the pitch length is unambiguously determined by the chirality, *ɛ*_3_. Thus, fixing the latter causes helices to form that are characterized by a fixed pitch length regardless of whether these helices are part of a cholesteric or a blue-phase structure. From the plot in Figure 4, it is easy to determine the optimum substrate separation, such that a helix of an integer half-pitch can be accommodated, which is not strained by the mismatching substrate separation. If one now moves away from this optimum substrate separation by making *s_z_* larger or smaller, the helix is exposed to a compressional/dilatational strain, even though the original half-integer pitch is not immediately disrupted. If the compressional/dilatational strain exceeds a certain threshold, the helix can no longer withstand the strain, and a transition to an ordinary nematic phase is observed. This is illustrated by the snapshots in Figure 9a,b, where, starting from *s_z_* = 12.5 (see Figure 9a), an originally cholesteric phase is disrupted by the compressional stress associated with a reduction of the substrate separation to *s_z_* = 8.2 (see Figure 9b), where a conventional nematic phase is observed.

A more interesting case is observed at a substrate separation, *s_z_* = 21.3, which is large enough, such that a double-twist helix of one full pitch can be accommodated between the confining surfaces and our choice of *ɛ*_3_ = 0.24. At both substrates, molecules are anchored directionally [see Equation (10a)] such that their longer axes are preferentially aligned with the *x*-axis. Employing grand-canonical simulations, we are able to screen different *x*–*y* side lengths around one pitch length and observe, consistently, the same structure, which will be described in the following. Because our present choice of *ɛ*_3_ = 0.24 slightly favors the formation of a blue phase and the directional anchoring would favor formation of a cholesteric phase in which the director rotates around the *z*-axis, one anticipates a competition between the double-twist helix characteristic of a blue phase and a homogeneous director field in the *x*–*y* plane, indicative of a cholesteric phase. Indeed, an inspection of the snapshot presented in Figure 9c illustrates the formation of both structures side by side in the same simulation.

**Figure 10:**
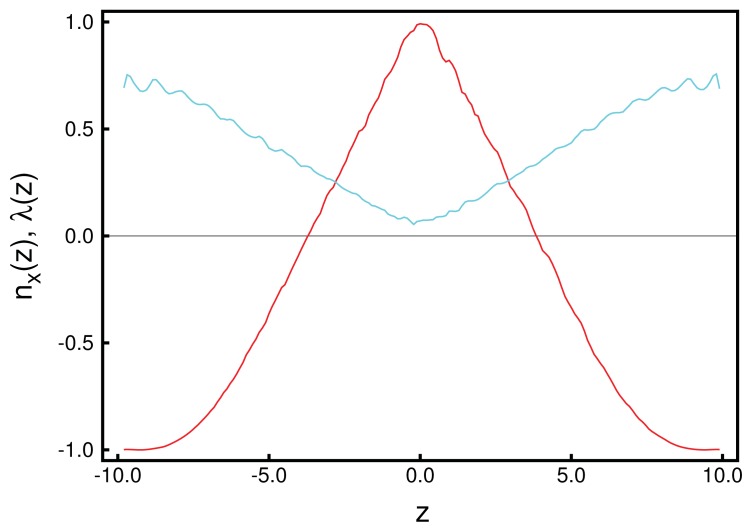
As Figure 1, but for *n_x_* (*z*) ( 
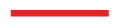
) and *λ* (*z*) ( 
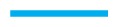
) and *ɛ*_3_ = 0.24.

**Figure 11:**
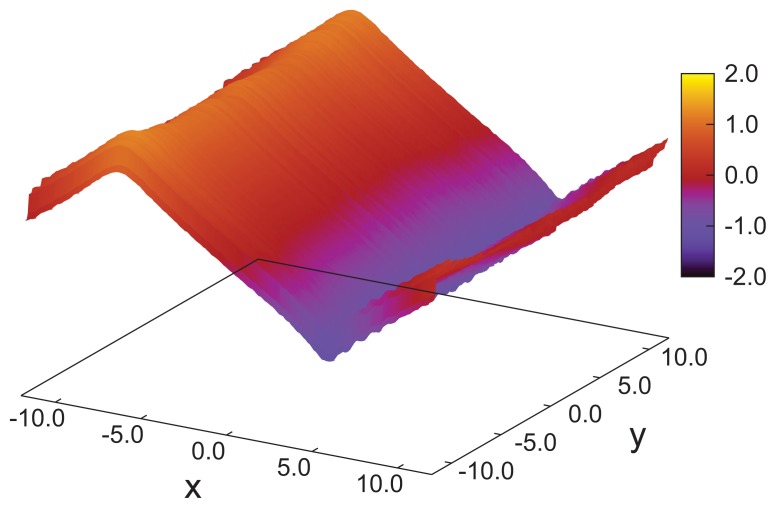
As Figure 2, but for *ɛ*_3_ = 0.24. One can see the periodic structure in the *x* direction and the constant behavior in the *y* direction.

A more quantitative analysis of this peculiar new structure is presented in Figure 10, which shows that there is a substantial decrease of the local nematic order parameter, *λ* (*z*), as one approaches the center of the simulation cell at *z* = 0. This is a result of the compressional/dilatational strain mentioned before. At the same time, one notices that *n_x_* (*z*) varies periodically, corresponding to a full-pitch helical structure. However, a comparison with the plots in Figure 1 shows that for the present case, the variation of *n_x_* (*z*) cannot be described by a sine function, but exhibits a peculiar triangular shape. An even more detailed picture emerges from the plot of *n_z_* (*x*) + *n_z_* (*y*) presented in Figure 11. Here, one notices the periodic variation of *n_z_* (*x*) + *n_z_* (*y*) along the *x*-axis, reminiscent of the double-twist helix forming along that axis (see also Figure 7) and that *n_z_* (*x*) + *n_z_* (*y*) = const along the lines of *x* = const, which was a feature of cholesteric-like structures (see Figure 2).

The mixed new structure illustrated by plots in Figures 9, 10 and 11 is a result of a rather complex interplay between confinement, chirality and anchoring at the substrates. First, chirality slightly favors the formation of double-twist helices, according to the plot in Figure 4. However, this requires mesogens to point towards the substrates as one moves out of the center of the double-twist helix in any radial direction. This orientation is energetically unfavorable with respect to those portions of the liquid crystal anchored at the substrates along the *x*-axis. In fact, these mesogens would like to align their neighbors in a twisted, but otherwise in-plane arrangement, such that a cholesteric phase would result with a helix forming around the *z*-axis. We believe that on account of thermal fluctuations, sometimes the cholesteric-like structure wins in a certain part of the system, whereas in another region and at a different stage of the evolution of our system, the double-twist helical structure may be favored.

**Figure 12:**
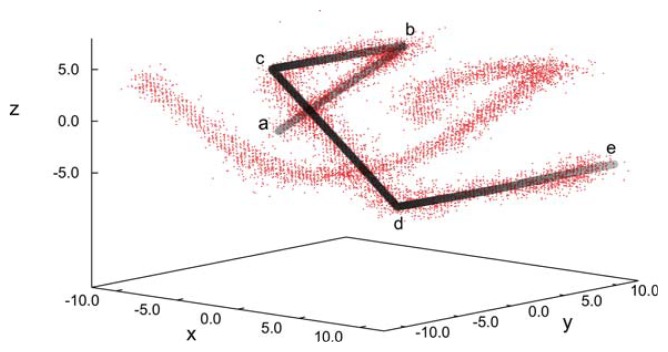
As Figure 8, but for the confined phase. Black/grey lines are employed to guide the eye. Characteristic points along one disclination line are marked by letters a–e (see text).

The uniqueness of the new structure observed under special confinement, chirality and anchoring conditions is also illustrated by the spatial variation of disclination lines, which we define according to the same criterion already introduced above with respect to our analysis of blue phase II. The plot of disclination lines in Figure 12 exhibits two independent such lines that cross one another, but never join. Starting at point “a” in Figure 12, the marked disclination line first varies along the *x*-axis and, thereby, approaches the upper substrate at point “b”. It then turns rather abruptly and runs along the *y*-axis in the negative direction. At point “c”, the disclination line exhibits another sharp turn into the *x*-direction and descends to the plane of the lower substrate. Once that approach of the lower substrate commences at point “d” the disclination line turns again into the *y*-direction and remains at constant *z*, ending at point “e”, which is is equivalent to point “a”, due to periodic boundary conditions. The other disclination line plotted in Figure 12 shows the corresponding spatial variation, as we have verified by visual inspection of the curves under different angles. This structure is distinctly different from the plot in Figure 8b for blue phase II and, also, from the spatial variation one would expect for blue phase I [20].

## 5. Conclusions

We investigated the formation of cholesteric and blue phase II in a new model for chiral liquid crystals by means of MC simulations in a specialized isothermal-isobaric and a grand canonical ensemble. Through specific anchoring conditions at the planar substrates of a slit-pore, we are able to determine the pitch length as a function of the chirality parameter, *ɛ*_3_, via the nematic director. The latter rotates along one or several axes of the Cartesian coordinate system in the cholesteric and blue phase, respectively. We determine the pitch length over a wide range of chiralities ranging from *ɛ*_3_ = 0.08 to *ɛ*_3_ = 0.9. Knowing the pitch length, we can set up systems that are not exposed to spurious strains, due to periodic boundary conditions, because the side lengths of our simulation cell can be taken as half-integer multiples of the pitch length. Because of this setup, we are able to observe the undisturbed helical structure of blue phases in all three spatial dimensions and observe the same pitch length for each as one would expect. If the pitch length is large enough, we can also visualize the disclination lines characteristic of blue phase II. We emphasize that in our model, chirality has to be sufficiently large to obtain a well-defined blue phase. According to typical snapshots presented in Figure 6, the size of double-twist helices forming under conditions of our simulations may seem somewhat small compared with experimental observations. This model intrinsic property turned out to be useful, because it permits us to investigate systems accommodating several pitches with a reasonable number of particles that is not too large. Nevertheless, it should be realized that these conditions are somewhat unrealistic with regard to typical experimental pitch lengths. However, we stress that despite this mismatch, all structural features, such as the simple-cubic unit cell and the geometry of disclination lines, fully comport with those known for a blue phase II, so that we believe our model system to be sufficiently realistic.

By choosing a certain chirality parameter, *ɛ*_3_ = 0.24, we also investigated the interplay between confinement, chirality and anchoring conditions at the solid substrates. We could show that even for just a single chirality value, three different phases are possible, the nematic, the cholesteric and a novel confined phase, which may be perceived as a result of a competition between ordinary cholesteric and blue phases. Unlike the latter two, the new phase is characterized by the formation of a helical structure in two dimensions rather than one (cholesteric phase) or three spatial directions (blue phase). Moreover, the new phase in confinement is characterized by a spatial variation of disclination lines that have not been observed before.
